# Plasma-Modified, Epitaxial Fabricated Graphene on SiC for the Electrochemical Detection of TNT

**DOI:** 10.3390/s16081281

**Published:** 2016-08-12

**Authors:** Scott A. Trammell, Sandra C. Hernández, Rachael L. Myers-Ward, Daniel Zabetakis, David A. Stenger, D. Kurt Gaskill, Scott G. Walton

**Affiliations:** U.S. Naval Research Laboratory, Washington, DC 20375, USA; hernandez.hangarter@nrl.navy.mil (S.C.H.); rachael.myers-ward@nrl.navy.mil (R.L.M.-W.); daniel.zabetakis@nrl.navy.mil (D.Z.); david.stenger@nrl.navy.mil (D.A.S.); gaskill@nrl.navy.mil (D.K.G.); scott.walton@nrl.navy.mil (S.G.W.)

**Keywords:** plasma modified graphene, epitaxial graphene, electrochemical detection, square wave voltammetry

## Abstract

Using square wave voltammetry, we show an increase in the electrochemical detection of trinitrotoluene (TNT) with a working electrode constructed from plasma modified graphene on a SiC surface vs. unmodified graphene. The graphene surface was chemically modified using electron beam generated plasmas produced in oxygen or nitrogen containing backgrounds to introduce oxygen or nitrogen moieties. The use of this chemical modification route enabled enhancement of the electrochemical signal for TNT, with the oxygen treatment showing a more pronounced detection than the nitrogen treatment. For graphene modified with oxygen, the electrochemical response to TNT can be fit to a two-site Langmuir isotherm suggesting different sites on the graphene surface with different affinities for TNT. We estimate a limit of detection for TNT equal to 20 ppb based on the analytical standard S/N ratio of 3. In addition, this approach to sensor fabrication is inherently a high-throughput, high-volume process amenable to industrial applications. High quality epitaxial graphene is easily grown over large area SiC substrates, while plasma processing is a rapid approach to large area substrate processing. This combination facilitates low cost, mass production of sensors.

## 1. Introduction

Being all surface, graphene is an excellent material to sense adsorbates as these can alter the charge carrier concentration, leading to measurable changes in conductivity. Schedin et al. [1], exploited this property by demonstrating detection sensitivity due to single molecule events using graphene flakes. Since large-area graphene is required for realistic applications, graphene formed by the sublimation of Si from SiC (0001) wafer surfaces is attractive for sensor development due to ease of fabrication and obvious manufacturability [2]. Using the epitaxial graphene approach, sensitive molecular sensors for many different analytes, i.e., adsorbates, have been successfully demonstrated by others [3,4,5]. However, this natural sensitivity to a range of analytes is also a disadvantage and thus sensors should be designed to minimize or discriminate against the effects associated with non-target constituents in the operating ambient conditions [6,7]. Chemical functionalization is a means to achieve this. Square wave voltammetry as an electroanalytical technique appears attractive as a discriminating sensor platform, and, moreover, epitaxial graphene has successfully been used as the working electrode; [8] but the near-perfect crystallinity of epitaxial graphene [9,10] is expected to reduce sensitivity for this approach due to the limited number of electrochemically active (oxidation/reduction) sites [11]. Recently, it was implied that reaction sites can be controllably formed on epitaxial graphene as it was demonstrated that plasma-based, oxygen functionalization of epitaxial graphene enhanced the molecular detection sensitivity for planar device geometries as well as greatly improving sensor response time [12]. For these reasons we have undertaken an investigation using controllably functionalized graphene as the electrode in electroanalytical sensing applications.

Carbon nanomaterials have recently received attention in electroanalytical sensing applications with examples in implantable sensors [13], ion sensors for point-of-care analysis [14], and trace analysis of heavy metals [15]. In this work, our target analyte is trinitrotoluene (TNT) as sensitive sensors for this and similar substances are of general interest. Analytical techniques for TNT determination include fluorescence immunoassays [16], fluorescence quenching [17], chromatography [18] and Raman spectrometry [19]. However, electroanalytical techniques have an advantage since the instrumentation is simple and can be miniaturized for field application [20,21]. In addition, nitro-aromatics are well known to give unique signature voltammograms for identification [22].

Previous work on graphene-based electrochemical detection of nitroaromatic substances used a variety of preparation methods utilizing graphene or reduced graphene-oxide as a working electrochemical sensor and reports that limits of detection (LOD) for TNT range from 0.2 ppb to 100 ppb [23,24,25,26,27,28]. In most cases, achieving low LODs regularly requires an electrochemical accumulation step where an applied voltage at the working electrode is used to accumulate TNT at the surface of the electrode [29]. In addition, the preparation methods for these approaches are often limited in scalability, making practical application on the industrial scale intractable. Limits of detection for other forms of carbon include glassy carbon (LOD = 130 ppb) [30] and screen printed carbon (LOD = 200 ppb) [31].

In this manuscript, we show that plasma modified [32] epitaxial graphene [33] shows improved electrochemical detection of TNT (LOD ≈ 20 ppb) without the need for an accumulation step [29], or preconcentration step that utilizes large volume samples to provide sufficient analyte mass for obtaining a detectable signal [34], or an amplification step in which the TNT signal is enhanced using an electrochemical technique termed redox cycling [35], all to improve the electrochemical response.

## 2. Materials and Methods

### 2.1. Growth of Epitaxial Fabricated Graphene on SiC

The epitaxial graphene was synthesized by means of Si sublimation from semi-insulating (SI), Si-face, on-axis, 6H-silicon carbide (SiC) substrates [36]. The synthesis took place in a commercial chemical vapor deposition reactor at a temperature of 1540 °C and a pressure of 100 mbar under an Ar ambient. The Ar was used to suppress the sublimation of Si in order to control the thickness of the epitaxial graphene layers. Prior to growth, the substrate was in-situ H_2_ etched to prepare the SiC surface for epitaxial graphene growth by removing any polishing scratches created during the manufacturing of the SiC substrate and forming bilayer stepped morphology. After growth, the sample was cooled in Ar to 800 °C, at which point the reaction tube was evacuated. The thickness of the epitaxial graphene layers was ≈2 monolayers as determined by X-ray photoelectron spectroscopy (XPS) using a Thermo Scientific K-Alpha spectrometer with a monochromatic Al-Kα source using a 400 µm spot size. Chemical analysis was performed using Avantage.

### 2.2. Plasma Functionalization

The epitaxial graphene was chemically modified using electron-beam generated plasmas, which are well-suited for large area plasma processing of atomically thin materials [36]. A detailed description of the processing system and protocols followed here, to introduce and control the relative concentration functional groups on the surface of graphene, can be found in earlier works [37,38,39]. Briefly, pulsed high-energy electron beams were produced by applying a −2 kV pulse to a linear hollow cathode with a −2 kV square wave for a duration of 1 ms at a duty factor of 10%. The emergent beam passes through a slot (1 × 20 cm^2^) in a ground anode, and terminates at a second grounded anode located further downstream (50 cm). The slotted anode defines the beam cross section and the volume between these two anodes defines the processing region. The electron beam is magnetically confined to minimize spreading via collisions with the background gas, producing a sheet-like plasma in the processing region. Substrates (graphene) are placed on a processing stage located 2.5 cm from the electron-beam axis. After evacuating the processing reactor to base pressure (~1 × 10^−6^ Torr), reactive gases, either O_2_ or N_2_ depending on the desired functionalities, are introduced at 5% of the total flow rate with argon providing the balance to achieve a target operating pressure. To vary the functional group density on the surface of epitaxial graphene [32], operating pressures were varied from 25 to 90 mTorr by controlling the total flow rate 50 to 180 sccm (standard cubic centimeter per minute). The total plasma processing time was 60 s.

### 2.3. Surface Characterization 

Ex-situ surface diagnostics were performed before and immediately after plasma processing to determine the starting material quality and chemistry and the changes resulting from plasma modification. Chemical changes and the resulting bonding characteristics in the graphene due to plasma processing were tracked by XPS. Chemical analysis was performed using Avantage and Unifit softwares.

### 2.4. Electrochemistry and Cell Design

Electrochemical measurements were performed using a potentiostat model #660 from CH Instruments. An electrochemical cell was composed of three electrodes (working, counter (Pt wire), and reference (Ag/AgCl)) all immersed in (or in contact with) an aqueous electrolyte solution. The working electrode was the epitaxial graphene, as received or chemically-modified with an approximately projected area of 1.6 mm^2^. Since the current signal generated in electrochemical measurements is proportional to the area of the working electrode, it was advantageous to restrict the active electrode to a well-defined area. When working electrodes are manufactured from rigid substrates, it is practical to form a cell on top of the electrode using an open-ended container and a gasket to prevent leaks. In order to form such a cell and to allow for the electrical connection to the graphene/SiC substrate, a custom cell was designed. The cell contains an integrated gasket, a broad base for clamping, and a cut-out to allow maximum access to the working electrode. The reference and counter electrodes were suspended in the upper funnel-shaped well which also holds the electrolyte.

## 3. Results

### 3.1. Plasma Functionalization and Surface Characterization

**Figure 1:**
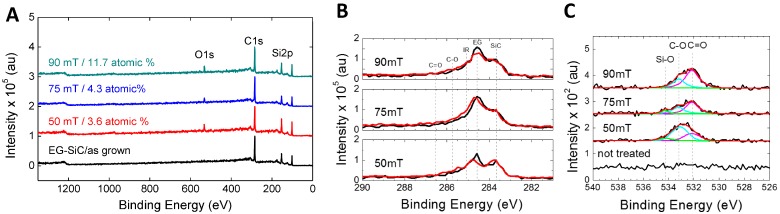
X-ray photoelectron spectroscopy (XPS) of unmodified, and oxygen modified graphene, depicting the (**A**) survey spectra; (**B**) the high resolution C1s spectra and (**C**) the high resolution O1s spectra. The black curve represents before functionalization, and red curve after functionalization in (**B**).

Electron beam generated plasmas produced in O_2_/Ar mixtures resulted in the introduction of oxygen functionalities on the surface of graphene. Shown in Figure 1A are the XPS spectra of as-grown and plasma-modified samples, processed at different operating pressures (50–90 mTorr). Following plasma exposures, the presence of oxygen is clear (O1s) and shows a gradual increase in the total oxygen content with increasing operating pressure, going from 3.6% at 50 mTorr to 11.7% at 90 mTorr. A better understanding of the functionalization can be developed by comparing the high-resolution spectra of graphene before and after processing for each plasma processing condition. The results are shown in Figure 1B. The C 1s peaks for untreated EG is composed of three components centered at about 283.6 eV, 284.5 eV and 285.3 eV which correspond to the silicon carbide substrate (Si–C), the epitaxial graphene film (C–C), and the interfacial layer between the SiC and epitaxial graphene, respectively [6]. Here, the graphene peak with sp^2^ bonding is labeled as EG, the interfacial layer is labeled as IR, and the silicon carbide signal is referred to as SiC. The spectra presented in Figure 1B have been shifted to the intensity of the substrate (Si–C) peak at ≈283.6 eV. Additionally, C1s spectra shows the incorporation of oxygen functionalities which are assigned to carbon bonding involving ethers or alcohols (C–O–C, C–O, or C–OH) and carbonyl bonds (=O) located at ≈286.4 eV and ≈287.1 eV, respectively. Before plasma processing, the O1s scans (Figure 1C) show little to no signal, indicating no oxygen present on the samples. After plasma processing, features on the O1s spectra arise at three different locations corresponding to (Si–O) bonding at ≈534.3 eV, ethers or alcohols (C–O–C, C–O, or C–OH) at ≈533.3 eV and carbonyl groups (C=O) at ≈532.2 eV. The de-convolved O1s spectra shows pressure dependence in terms of the amount and type of carbon-oxygen bonds formed in the O_2_/Ar plasma [32].

**Figure 2:**
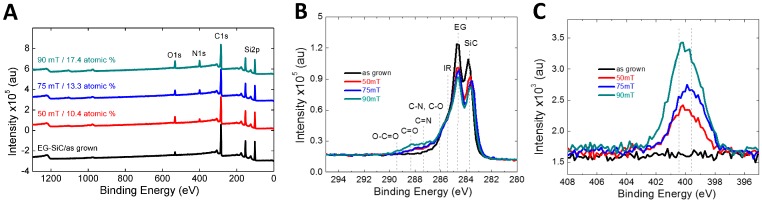
XPS of unmodified, and nitrogen modified graphene, depicting the (**A**) survey spectra; (**B**) the high resolution C1s spectra and (**C**) the high resolution N1s spectra. The dotted vertical lines represent approximate peak positions.

Similarly, nitrogen functionalities can be introduced in a controlled manner using electron beam generated plasmas produced in an N_2_/Ar mixture at various operating pressures as seen in Figure 2A. The assignments of the C1s components are challenging due to the overlapping binding energies of nitrogen and oxygen species with those of the interfacial layer. However, based on the combined features of the C1s (Figure 2B) and N1s (Figure 2C) high resolution spectra, the identifiable peaks located at ≈283.6 eV, 284.5 eV, 285.3 eV, are attributed to the Si–C, C–C sp^2^, and interfacial layer, respectively. The peaks located at higher binding energies (>286 eV) are attributed to nitrogen and oxygen functionalities. Notably C–N and C–O a ≈286.1 eV, C=N at ≈287.4 eV, C=O and N–C=O at ≈288.3 eV, and O–C=O at ≈289.4 eV. The N1s spectra indicates that nitrogen functionalities are present primarily in the amide and pyrolic configurations.

### 3.2. Electrochemistry of TNT at Modified Graphene

The electrochemical response of the reduction of TNT at the graphene working electrode was characterized using square-wave voltammetry, which is a common electroanalytical technique for analytical applications since it diminishes non-faradaic charging currents that develop at the solution electrode interface when employing potential sweeping techniques [40]. In square-wave voltammetry, a potential staircase is overlaid on the voltage ramp as the voltage is swept in the desired range at the working electrode. The reduction (or oxidation) of the analyte occurs as the voltage approaches its formal redox potential, the potential of the first minimum is the first reduction, named the cathodic peak potential, *E_pc_*. The current is then measured at different points of the potential waveform to minimize capacitive charging at the solution electrode interface. Since non-faradic current decays faster than faradic current [40], a square-wave voltammogram can be generated by taking the difference of current between the points subtracting out the capacitive charging current making the detection limits typically better then cyclic voltammetry. In this way, the maximum absolute value of the measured current or the net cathodic peak current, *I_pc_*, is determined (measured with respect to the background current taken without TNT) at *E_pc_*.

**Figure 3:**
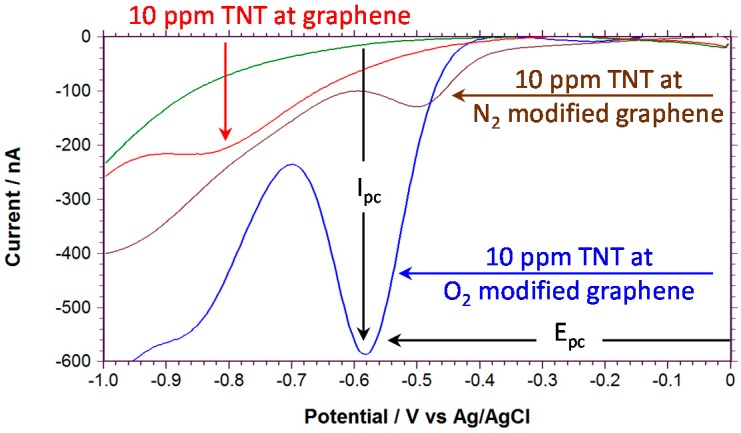
Square wave voltammetry 10 ppm of TNT in air saturated phosphate buffered saline (PBS) buffer measured at a fabricated epitaxial graphene working electrode on SiC (red), with oxygen modification (90 mTorr) (blue) and with nitrogen modification 50 mTorr (brown). The green trace is the background signal without TNT. Frequency = 60 Hz and Amplitude = 25 mV.

To record the square wave voltammograms, the modified graphene samples were mounted in a custom built electrochemical cell and background measurements were recorded before the addition of TNT. Representative examples of the square wave voltammetry for the reduction of 10 ppm of TNT at these modified graphene samples are shown in Figure 3. The square wave voltammograms were measured in air-saturated phosphate buffered saline (PBS, pH 7.4; pH did not change with TNT admixtures) solutions from 0 to −1.0 V vs. Ag/AgCl reference electrode. With unmodified graphene, the first reduction of TNT (*E*_pc_ = −0.8 V vs. Ag/AgCl) is shifted to significantly more negative voltages compared to oxygen functionalized graphene (*E*_pc_ = −0.58 V vs. Ag/AgCl) and nitrogen functionalized graphene (*E*_pc_ = −0.5 V vs. Ag/AgCl) consistent with a similar report [41]. In comparison, other carbon electrodes such as glassy carbon show the first reduction near −0.575 V vs. Ag/AgCl [34]. In square wave voltammetry, changes in *E_pc_* and *I_pc_* are a reflection of both thermodynamic and kinetic properties of the electrochemical system, hence these oxygen functionalized working electrode results indicate that functionalization either increases the active surface area of the graphene or increases the rate of reduction of the TNT or both. The more negative *E_pc_* would decrease the resistance to interference with oxygen.

**Figure 4:**
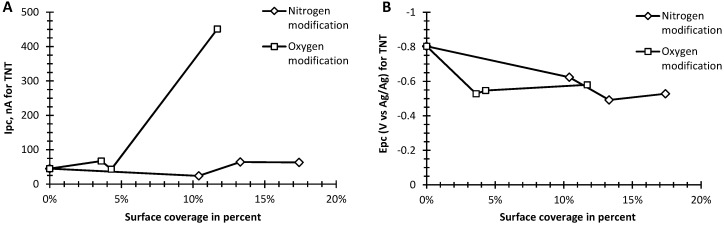
(**A)** Net cathodic peak current (*I*_pc_) and (**B**) cathodic peak potential (*E*_pc_) vs. surface coverage in percent of nitrogen (◊), and oxygen (▯) with bare graphene at 0%.

In Figure 4, *I*_pc_ and corresponding *E*_pc_ are shown for a 10 ppm sample of TNT in PBS vs. nitrogen or oxygen functionalized content of the graphene samples. Results for unfunctionalized graphene, are given for 0% content. Figure 4A shows that for the nitrogen functionalized samples there is little change to *I_pc_* with increasing content, however, *E_pc_* becomes less negative with increasing nitrogen content and begins to saturate at 13 atomic % (Figure 4B). For oxygen functionalized samples, the oxygenated graphene *I_pc_* of TNT is significantly enhanced, being 10-fold larger compared to nitrogen functionalized samples as can be seen in Figure 4A. Yet, *E_pc_* also becomes less negative with increasing content and saturates earlier, near 4 atomic %, than for the nitrogen functionalized samples. These results imply the oxygen functionalized samples should have increased TNT detection sensitivity.

### 3.3. Trace Detection of TNT for Oxygen Modified EG

**Figure 5:**
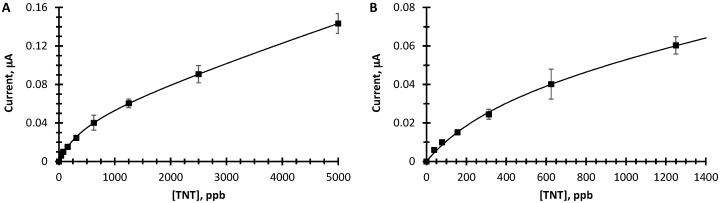
Dose response curves for oxygen functionalized epitaxial graphene. (**A**) Net peak amplitude, I_pc_ vs. {TNT}, for high ppb range; (**B**) Net peak amplitude vs. {TNT} for low concentration. The line is a least squares fit of the data to Equation 1 with a = 0.047, b = 3.7, K_a_ = 0.05 µM^−1^, K_b_ = 1.3 × 10^−4^ µM^−1^, (K units has been converted to µM^−1^) range.

We quantified the increased TNT detection sensitivity for oxygen functionalized graphene by acquiring square-wave voltammograms for various concentrations of TNT in PBS. Figure 5 shows the results of these measurements where Figure 5A displays a larger TNT concentration range (0 to 5000 ppb) than 5B (0 to 1400 ppb). It is readily apparent that the oxygen functionalized graphene yields sensitivities spanning over 100-fold. In addition, dependence on TNT concentration can be fit to a two-site Langmuir isotherm (Equation (1)) suggesting different active sites with different TNT affinities on the graphene surface.

Net peak amplitude = a(K_a_[TNT])/(1 + (K_a_ [TNT])) + b(K_b_[TNT])/(1 + (K_b_[TNT])) (1)

In Equation (1), a is the proportion of current due to the reduction of TNT at sites a, K_a_ is the binding constant of TNT at sites a, b is the proportion of current due to the reduction of TNT at sites b, and K_b_ is the binding constant of TNT at sites b. The sites with high affinities for TNT saturate with increasing TNT concentration and the sites with lower affinities give rise to the large dynamic range. At 40 ppb (the lowest TNT concentration tested) the S/N ratio is 6. From this, we estimate a LOD of 20 ppb based on the analytical standard S/N ratio of 3.

## 4. Discussion

For carbon electrodes, surface preparation is often critical for good electrochemical response of analytes [42]. This has been shown when comparing basal plane to edge plane graphite, surface modification of glassy carbon, and more recently with different preparations of graphene and graphene oxides [29]. A common theme to describe the phenomenon is that electron transfer between the analyte and carbon surface occurs at defect sites, or chemical groups from the surface modification. In our case, the initial graphene synthesized on SiC gives a broad signal for TNT reduction at a more negative potential which suggests that graphene without any chemical modification has a slower electron transfer rate requiring a higher over potential to generate the TNT signal. Modification with oxygen and nitrogen both lower the over potential for the reduction of TNT. However, with oxygen modification, a significant increase in signal amplitude is shown. In this case, a two-site Langmuir isotherm can fit the dose response curve and suggests that oxygen modification generates several sites on the graphene surfaces with different affinities of TNT consistent with the range of functional moieties found in the XPS spectra.

## 5. Conclusions

We have shown a 6-fold increase in sensitivity towards the lower detection limit of TNT using chemically-modified graphene on a SiC surface compared to untreated graphene. The graphene surface was chemically modified using electron beam generated plasmas produced in oxygen- and nitrogen-containing backgrounds to introduce oxygen or nitrogen moieties. While both treatments enabled the enhancement of the electrochemical signal for TNT, oxygen-functionalized epitaxial graphene provided a more pronounced response. The oxygen functionalized graphene revealed a two-site Langmuir isotherm response extending over 2 orders of magnitude with a LOD of 20 ppb for TNT in PBS. The approach described here to fabricate graphene sensors sensitive to TNT is attractive for low cost, high-volume industrial production. This is because high quality epitaxial graphene is easily grown over large area SiC substrates and the plasma processing approach to oxygen functionalization is amenable to large area substrate processing.

## Abbreviations

The following abbreviations are used in this manuscript:

PBS	phosphate buffered saline
TNT	2,4,6-trinitrotoluene
EG	epitaxial graphene
